# Chronic prednisone, metformin, and nonsteroidal anti-inflammatory drug use and clinical outcome in a cohort of bladder cancer patients undergoing radical cystectomy in Québec, Canada

**DOI:** 10.1186/s12894-023-01287-6

**Published:** 2023-07-14

**Authors:** Michel D. Wissing, Ana O’Flaherty, Alice Dragomir, Simon Tanguay, Wassim Kassouf, Armen G. Aprikian

**Affiliations:** 1 Division of Urology, Department of Surgery, McGill University Health Center – Research Institute, 1001 Boulevard Decarie, D02.8100, Montreal, Québec H4A 3J1 Canada; 2 Department of Oncology, McGill University, 5100 Maisonneuve Blvd West, Suite 720, Montreal, Québec H4A 3T2 Canada

**Keywords:** Urinary bladder neoplasms, Survival, Radical cystectomy, Prednisone, Metformin, Anti-inflammatory agents, non-steroidal

## Abstract

**Background:**

Studies have suggested a positive association between bladder cancer (BC) outcome and comedication use, including nonsteroidal anti-inflammatory drugs (NSAID), metformin, and prednisone use. To validate these associations, we evaluated whether these medications were associated with clinical outcome in a Canadian cohort of BC patients.

**Methods:**

This is a retrospective cohort study on BC patients undergoing radical cystectomy (RC) in Québec province in 2000–2015, as registered in the provincial health administration databases. Medication use was considered chronic when prescribed for ≥ 1 year. Overall (OS), disease-specific (DSS) and recurrence-free (RFS) survival were compared using multivariable Cox proportional hazards models. Covariates included age, Charlson’s comorbidity index, region of residence, year of RC, distance to hospital, hospital type, hospital and surgeon annual RC volume, neoadjuvant chemotherapy use, and type of bladder diversion, as well as mutual adjustment for concomitant comedication use (statins, NSAIDs, metformin, and prednisone).

**Results:**

Of 3742 patients included, 293, 420, and 1503 patients chronically used prednisone, metformin, and NSAIDs before surgery, respectively. In multivariable analyses, preoperative prednisone use was associated with improved OS (HR 0.67, 95%CI 0.55–0.82), DSS (HR 0.58, 95%CI 0.45–0.76), and RFS (HR 0.61, 95%CI 0.47–0.78). Patients who chronically used metformin preoperatively had a worse OS (HR 1.29, 95%CI 1.07–1.55), DSS (HR 1.38, 95%CI 1.10–1.72), and RFS (HR 1.41, 95%CI 1.13–1.74). Preoperative, chronic NSAID use was not significantly associated with all clinical outcomes, with adjusted HRs for OS, DSS, and RFS of 1.10 (95%CI 0.95–1.27), 1.24 (95%CI 1.03–1.48), and 1.22 (95%CI 1.03–1.45), respectively. Directionality of findings was similar when stratifying by comedication use in the year following surgery. Results were similar after propensity-score matching too.

**Conclusions:**

In our Canadian cohort of BC undergoing RC, chronic prednisone use was associated with improved clinical outcomes, while metformin and NSAID were not.

**Supplementary Information:**

The online version contains supplementary material available at 10.1186/s12894-023-01287-6.

## Introduction

Bladder cancer is the 5th most incident solid cancer in the United States and Canada [1, 2]. Due to the increased bladder cancer incidence in older patients, most bladder cancer patients will have comorbidities requiring medication. Several frequently used medications have been suggested to affect bladder cancer incidence and outcome [3]. It is important to study such associations.

In Québec province in Canada, we have linked several provincial administrative datasets to create a cohort of patients undergoing radical cystectomy (RC) for bladder cancer between 2000 and 2015 [4]. Using this cohort, we previously identified an association with chronic statin use and improved survival in bladder cancer patients [5], but could not confirm earlier reports that 5α-reductase inhibitors and α_1_-blockers may be associated with improved bladder cancer outcome [6].

NSAIDs [3], including aspirin [7, 8], and metformin [9] are other frequently prescribed medications that have been associated with bladder cancer outcome, all seemingly having a protective effect. Others have disputed the protective effects of these medications [10, 11]. As these conclusions are based on observational clinical studies, various biases may have contributed to such discrepancies, such as selection, publication, and immortal time biases [12]. Hence, we aimed to validate associations between NSAIDs and metformin use in our large bladder cancer cohort. Additionally, we evaluated whether prednisone use was associated with bladder cancer outcome. Considering that it is thought that the protective effect of NSAIDs is due to its anti-inflammatory effect, we hypothesized that stronger anti-inflammatory drugs such as prednisone would further improve bladder cancer outcome. Furthermore, prednisone has been used in various combination therapies against certain cancers, such as prostate cancer [13–16] and leukemia [17], and some preclinical studies have suggested that prednisone may inhibit bladder cancer progression as well [18]. As such, our study is the first clinical cohort study evaluating whether prednisone use is associated with improved clinical outcomes in bladder cancer patients.

## Patients and methods

### Study design, cohort selection, and data acquisition

This is a retrospective cohort study of bladder cancer patients undergoing RC by a urologist in Québec province, Canada, between 2000 and 2015. Data were collected by combining three Québec provincial administrative databases:

- RAMQ, which collects all public medical and pharmaceutical services provided to patients in the province;
- ISQ, which collects death statistics on Québec inhabitants;
- MED-ÉCHO, which collects data on hospitalization of patients in public hospitals in the province.

We previously described in detail how we created this cohort, using various restrictions to ensure the cohort was limited to patients undergoing RC for bladder cancer [4]. Since Québec province has a universal health care system, virtually all RCs are conducted in public hospitals and therefore collected by these databases.

Aforementioned databases registered all medical procedures performed in public medical centers in Québec, hospital and emergency room admissions and intensive care unit stays in the province, medication prescribed in outpatient pharmacies to patients covered by the Public Prescription Drug Insurance Plan, and deaths across the province. In Québec, healthcare insurance is mandatory, either via private healthcare insurance or the government-funded Public Prescription Drug Insurance Plan. RAMQ does not register medication use of those covered by private healthcare insurance. Therefore, patients with private healthcare insurance were excluded from our current analyses.

Patient data is encoded so that researchers with access to the database are unable to retrace the data to individual patients. Therefore, these databases cannot be linked to clinical data such as pathological tumor staging. We collected data from two years before radical cystectomy until December 31, 2016.

Similar to previous publications [5], patients were considered to be chronic, preoperative users of medication when the time between first and last prescription in our database was ≥ 365 days, and when the date of first prescription was before the date of RC. For postoperative medication use, we included all patients who had ≥ 1 prescription in the year following surgery. Controls were those who were never prescribed a specific medication, neither before nor after RC. As the available database did not allow calculation of the total dose of medication received, medication use was evaluated as a binary variable.

### Outcome parameters

Endpoints were recurrence-free, bladder cancer-specific, and overall survival. Detailed definitions of these outcome parameters have been provided previously [5, 6]. Date of RC was considered t = 0. Importantly, for recurrence-free survival, patients who had chemo- or radiotherapy within 90 days of postoperative hospital discharge were excluded as this was considered adjuvant or salvage therapy. For bladder cancer-specific survival, those who died within 90 days after surgery were excluded, to exclude patients who died from the direct consequences of surgery instead of the cancer.

A landmark analysis was conducted to eliminate immortal time bias in the evaluation of postoperative medication use, restricting our survival analyses to patients who survived ≥ 1 year while selecting patients based on their prescriptions in the first year following surgery.

### Statistical analyses

Stata (v15.1, Statacorp, College Station, Texas, USA) was used for all statistical analyses. Categorical baseline characteristics were reported as number of patients with percentages and were compared between groups using Chi-square tests. Continuous variables were displayed as the median with interquartile range and were compared using nonparametric Wilcoxon rank-sum tests. Median follow-up time was calculated using the reverse Kaplan-Meier method and displayed as the median with 95% confidence interval (CI) [19].

Survival analyses were conducted using the Kaplan-Meier method and log-rank tests [20]. Uni- and multivariable Cox proportional hazard models were used to calculate (adjusted) hazard ratios (HR) for survival [21]. Covariates in the multivariable analyses included: age (continuous variable), sex (dichotomous: male/female), region of residence (categorical: urban regions with > 400,000 inhabitants, (sub)urban regions with 100,000-400,000 inhabitants, and rural regions with < 100,000 inhabitants) [4], Charlson’s comorbidity index (continuous; values ranged between 2 and 18), year of surgery (dichotomous: 2000–2009 and 2010–2015), distance to the hospital by road (continuous), hospital type (dichotomous: academic/non-academic), hospital and surgeon average annual RC volume (continuous), type of bladder diversion (dichotomous: ileal conduit or continental diversion), administration of neoadjuvant chemotherapy (dichotomous: yes/no), and mutual adjustment for concomitant comedication use of statins, NSAIDs, metformin and prednisone (per medication, dichotomous: yes/no). These variables were selected as they affected clinical outcome in previous studies with the same cohort [4, 22]. Patients with missing data were excluded from multivariable analyses. A complete case analysis was used for all analyses, based on the variables of interest for the specific analysis.

For propensity score matching, patients were matched by all aforementioned variables except for concomitant co-medication use. For NSAID use, we matched 1:1 (with replacement) as approximately half of the patients were preoperative, chronic NSAID users. For metformin and prednisone use, we matched 1:3 with replacement, as less than 500 patients used these medications chronically before surgery. A caliper width of 0.2 was used for all analyses. Matching for concomitant medication use resulted in very low matching rates, creating a highly selective cohort. Instead, multivariable Cox proportional hazard models were used with concomitant medication use as a covariate.

## Results

In the provincial datasets, 4450 patients were identified who underwent RC for bladder cancer in Québec between 2000 and 2015. Of these, 708 patients had private healthcare insurance and were therefore excluded, bringing the total cohort size to 3742 bladder cancer patients with universal health coverage. In this cohort, 1503 (40.2%), 420 (11.2%), and 293 (7.8%) chronically used NSAIDs, metformin, and prednisone in the two years preceding RC, respectively (Table 1). Of the 1503 patients who chronically used NSAIDs preoperatively, 1144 used aspirin and 464 used other NSAIDs, with 105 patients using both (supplementary Table 1). Medication users were generally older, had a higher Charlson’s comorbidity index, and used more frequently other comedications (P < 0.001). While chronic NSAID and metformin users were more frequently male (P = 0.005 and P = 0.030, respectively), chronic prednisone users were more frequently female (P = 0.002). Chronic NSAID users lived more frequently outside cities (P = 0.008) and further from hospitals (P = 0.020); this difference was not observed in other chronic medication users. The percentage of chronic metformin users had increased between 2000 and 2009 and 2010–2015 (from 9.3% to 16.0%, P < 0.001). While the percentage of chronic NSAID users (59.9% versus 58.9%, P = 0.59) and prednisone users (11.4% versus 10.5%, P = 0.45) remained stable between 2000 and 2009 and 2010–2015, neoadjuvant chemotherapy was less frequently used in patients who had preoperative, chronic use of NSAIDs (4.9% versus 7.4%, P = 0.009) or prednisone (2.7% versus 6.5%, P = 0.011). Median follow-up of the whole cohort was almost 8 years (median 7.9 years, 95% CI 7.6–8.4 years).

**Table 1:** Baseline characteristics, by preoperative chronic medication use

	All patients	NSAID use			Metformin use			Prednisone use		
		Preoperative chronic use	No	P-value	Preoperative chronic use	No	P-value	Preoperative chronic use	No	P-value
Number of patients	3742	1503	1025		420	3052		293	2363	
Age, median (IQR)	70 (64–76)	72 (67–77)	69 (62–75)	**< 0.001**	72 (67–76)	70 (63–76)	**< 0.001**	72 (67–76)	70 (63–76)	**< 0.001**
Sex										
- Male	2827 (75.5%)	1167 (77.6%)	746 (72.8%)	**0.005**	334 (79.5%)	2278 (74.6%)	**0.030**	203 (69.3%)	1829 (77.4%)	**0.002**
- Female	915 (24.5%)	336 (22.4%)	279 (27.2%)		86 (20.5%)	774 (25.4%)		90 (30.7%)	534 (22.6%)	
Region of residence										
- Regions with cities with > 400,000 inhabitants	1453 (38.8%)	555 (36.9%)	434 (42.3%)	**0.008**	164 (39.0%)	1187 (38.9%)	0.95	99 (33.8%)	936 (39.6%)	0.14
- Regions with cities with 100,000-250,000 inhabitants	2012 (53.8%)	839 (55.8%)	524 (51.1%)		228 (54.3%)	1641 (53.8%)		173 (59.0%)	1258 (53.2%)	
- Rural regions (largest city in region < 100,000 inhabitants)	252 (6.7%)	106 (7.1%)	55 (5.4%)		26 (6.2%)	201 (6.6%)		20 (6.8%)	150 (6.3%)	
- Unknown	25 (0.7%)	3 (0.2%)	12 (1.2%)		2 (0.5%)	23 (0.8%)		1 (0.3%)	19 (0.8%)	
Charlson’s comorbidity index, median (IQR)	7 (5–8)	7 (6–9)	6 (5–7)	**< 0.001**	8 (7–10)	6 (5–8)	**< 0.001**	8 (6–9)	6 (5–8)	**< 0.001**
Year of surgery										
- 2000–2009	2203 (58.9%)	839 (55.8%)	561 (54.7%)	0.59	189 (45.0%)	1835 (60.1%)	**< 0.001**	169 (57.7%)	1308 (55.4%)	0.45
- 2010–2015	1539 (41.1%)	664 (44.2%)	464 (45.3%)		231 (55.0%)	1217 (39.9%)		124 (42.3%)	1055 (44.6%)	
Hospital characteristics										
- Distance to hospital in km, median (IQR)	20 (7–77)	20 (7–85)	17 (7–57)	**0.020**	20 (8–67)	19 (7–71)	0.74	26 (10–80)	19 (7–76)	**0.048**
- Hospital type, academic	1886 (50.4%)	729 (48.5%)	534 (52.1%)	0.08	197 (46.9%)	1559 (51.1%)	0.11	149 (50.9%)	1208 (51.1%)	0.93
- Hospital RC volume/year, median (IQR)	13 (8–32)	13 (7–32)	13 (7–32)	0.90	13 (7–32)	13 (8–32)	0.32	13 (8–32)	13 (8–32)	0.71
- Surgeon RC volume/year, median (IQR)	7 (5–13)	7 (5–13)	8 (5–13)	0.61	7 (5–10)	7 (5–13)	0.43	7 (5–13)	7 (5–13)	0.77
Type of bladder diversion										
- Ileal conduit	3046 (81.4%)	1269 (84.4%)	830 (81.0%)	**0.012**	371 (88.3%)	2465 (80.8%)	**0.001**	248 (84.6%)	1896 (80.2%)	0.12
- Continent diversion	544 (14.5%)	176 (11.7%)	155 (15.1%)		38 (9.0%)	456 (14.9%)		37 (12.6%)	376 (15.9%)	
- Unknown	152 (4.1%)	58 (3.9%)	40 (3.9%)		11 (2.6%)	131 (4.3%)		8 (2.7%)	91 (3.9%)	
Neoadjuvant chemotherapy	208 (5.6%)	74 (4.9%)	76 (7.4%)	**0.009**	23 (5.5%)	170 (5.6%)	0.94	8 (2.7%)	154 (6.5%)	**0.011**
Preoperative chronic medication use										
- Statins	1406 (37.6%)	939 (62.5%)	206 (20.1%)	**< 0.001**	308 (73.3%)	1001 (32.8%)	**< 0.001**	142 (48.5%)	826 (35.0%)	**< 0.001**
- NSAIDs	1503 (40.2%)	-	-	-	284 (67.6%)	1128 (37.0%)	**< 0.001**	159 (54.3%)	864 (36.6%)	**< 0.001**
- Aspirin	1144 (30.6%)	1144 (76.1%)	-	-	257 (61.2%)	814 (26.7%)	**< 0.001**	116 (39.6%)	652 (27.6%)	**< 0.001**
- Other NSAIDs	464 (12.4%)	464 (30.9%)	-	-	56 (13.3%)	382 (12.5%)	**< 0.001**	60 (20.5%)	249 (10.5%)	**< 0.001**
- Metformin	420 (11.2%)	284 (18.9%)	61 (6.0%)	**< 0.001**	-	-	-	32 (10.9%)	259 (11.0%)	1.00
- Prednisone	293 (7.8%)	159 (10.6%)	57 (5.6%)	**< 0.001**	32 (7.6%)	242 (7.9%)	0.58	-	-	-
Follow-up time (in years), median (95% CI)	7.9 (7.6–8.4)	7.3 (6.7–7.7)	6.5 (5.6–7.4)		5.4 (4.7–6.4)	8.1 (7.7–8.5)		8.2 (7.2–8.9)	6.6 (6.3–7.2)	

CI, confidence interval; IQR, interquartile range; NSAID, nonsteroidal anti-inflammatory drugs; RC, radical cystectomy

Preoperative, chronic use of prednisone was associated with improved clinical outcome in multivariable Cox proportional hazard models (Table 2 and supplementary Table 2). Adjusted HRs for overall, bladder cancer-specific, and recurrence-free survival were 0.67 (95% CI 0.55–0.82), 0.58 (95% CI 0.45–0.76), and 0.61 (95% CI 0.47–0.78), respectively, for those who chronically used prednisone in the two years preceding RC. For preoperative chronic statin users, these adjusted HRs were 0.80 (95% CI 0.69–0.93), 0.72 (95% CI 0.60–0.86), and 0.73 (95% CI 0.62–0.88), respectively, compared to never-statin users. Contrarily, chronic use of metformin prior to surgery was associated with a worse clinical outcome, adjusted HRs for overall, bladder cancer-specific, and recurrence-free survival being 1.29 (95% CI 1.07–1.55), 1.38 (95% CI 1.10–1.72), and 1.41 (95% CI 1.13–1.74), respectively. Adjusted HRs were 1.24 (95% CI 1.03–1.48) and 1.22 (95% CI 1.13–1.74) for bladder cancer-specific and recurrence-free survival in preoperative, chronic NSAID users, also suggesting a worse outcome. Separating NSAID use by aspirin or other NSAIDs, HRs generally remained above 1 in multivariable analyses as compared to those who never used aspirins or other NSAIDs, respectively, albeit differences were not significant (supplementary Table 2).

**Table 2:** Characteristics associated with overall, bladder cancer-specific, and recurrence-free survival

	Overall survival		Bladder cancer-specific survival		Recurrence-free survival	
	Univariable	Multivariable	Univariable	Multivariable	Univariable	Multivariable
Preoperative chronic medication use						
- Statins	**0.86** **(0.79–0.94)**	**0.80** **(0.69–0.93)**	**0.79** **(0.71–0.88)**	**0.72** **(0.60–0.86)**	**0.80** **(0.72–0.89)**	**0.73** **(0.62–0.88)**
- NSAIDs	0.95 (0.86–1.05)	1.10 (0.95–1.27)	0.93 (0.82–1.05)	**1.24** **(1.03–1.48)**	0.94 (0.84–1.06)	**1.22** **(1.03–1.45)**
- Metformin	**1.21** **(1.07–1.38)**	**1.29** **(1.07–1.55)**	**1.20** **(1.03–1.40)**	**1.38** **(1.10–1.72)**	**1.24** **(1.07–1.44)**	**1.41** **(1.13–1.74)**
- Prednisone	**0.74** **(0.63–0.86)**	**0.67** **(0.55–0.82)**	**0.59** **(0.48–0.72)**	**0.58** **(0.45–0.76)**	**0.64** **(0.53–0.77)**	**0.61** **(0.47–0.78)**
Age, per 5-year increment	**1.22** **(1.19–1.25)**	**1.21** **(1.15–1.27)**	**1.16** **(1.13–1.20)**	**1.19** **(1.12–1.26)**	**1.12** **(1.09–1.15)**	**1.13** **(1.07–1.19)**
Female sex (ref: male sex)	0.96 (0.88–1.06)	0.91 (0.78–1.06)	1.01 (0.90–1.13)	0.85 (0.71–1.03)	1.04 (0.93–1.16)	0.87 (0.73–1.04)
Region of residence (ref: Regions with cities with > 400,000 inhabitants)						
- Regions with cities with 100,000-250,000 inhabitants	0.96 (0.89–1.05)	0.94 (0.81–1.09)	0.93 (0.873–1.03)	0.90 (0.75–1.08)	**0.90** **(0.82–0.99)**	**0.84** **(0.70-1.00)**
- Rural regions (largest city in region < 100,000 inhabitants)	0.94 (0.80–1.12)	1.18 (0.78–1.79)	0.96 (0.78–1.18)	1.23 (0.76–2.01)	0.91 (0.75–1.11)	1.00 (0.62–1.62)
Charlson’s comorbidity index, per 1 increment	**1.02** **(1.01–1.04)**	**0.96** **(0.93-1.00)**	**0.98** **(0.96-1.00)**	**0.93** **(0.88–0.97)**	**0.97** **(0.95–0.99)**	**0.93** **(0.89–0.98)**
Year of surgery, 2010–2015 (ref: 2000–2009)	1.02 (0.93–1.12)	**0.69** **(0.60–0.80)**	1.03 (0.92–1.14)	**0.67** **(0.57–0.80)**	1.05 (0.95–1.16)	**0.70** **(0.60–0.83)**
Hospital characteristics						
- Distance to hospital in km, per 50 km increment	1.00 (0.99–1.02)	1.00 (0.96–1.04)	1.01 (0.99–1.03)	1.00 (0.95–1.05)	1.01 (0.99–1.02)	1.01 (0.96–1.06)
- Academic hospital (ref: non-academic hospital)	**0.90** **(0.83–0.97)**	0.94 (0.78–1.13)	0.93 (0.85–1.03)	0.95 (0.75–1.19)	0.96 (0.87–1.05)	1.00 (0.80–1.24)
- Hospital RC volume, per 5/year increment	0.99 (0.98-1.00)	1.00 (0.96–1.04)	0.99 (0.98–1.01)	1.01 (0.96–1.06)	1.00 (0.98–1.01)	1.21 (0.96–1.05)
- Surgeon RC volume, per 5/year increment	**0.95** **(0.92–0.99)**	0.98 (0.88–1.09)	0.97 (0.93–1.01)	0.98 (0.86–1.11)	0.98 (0.94–1.02)	0.97 (0.86–1.10)
Continent bladder diversion (ref: ileal conduit)	**0.50** **(0.44–0.58)**	**0.58** **(0.46–0.73)**	**0.57** **(0.49–0.67)**	**0.61** **(0.47–0.80)**	**0.62** **(0.53–0.72)**	**0.62** **(0.48–0.79)**
Neoadjuvant chemotherapy	0.94 (0.77–1.15)	1.17 (0.87–1.58)	1.15 (0.92–1.44)	1.38 (1.00-1.91)	1.18 (0.96–1.46)	1.30 (0.95–1.78)

Uni- and multivariable Cox proportional-hazards regression models were used to calculate hazard ratios and their respective 95% confidence intervals. In the multivariable analyses, all variables listed were mutually adjusted for each other. Hazard ratios with a p-value of less than 0.050 are marked in bold

NSAID, nonsteroidal anti-inflammatory drugs; RC, radical cystectomy

In line with previous reports on our cohort, age was associated with a worse clinical outcome, while a higher Charlson’s comorbidity index, more recent surgery, and a continent bladder diversion were significantly associated with improved clinical outcomes after adjusting for comedication use and other potentially confounding variables (Table 2).

**Fig. 1:**
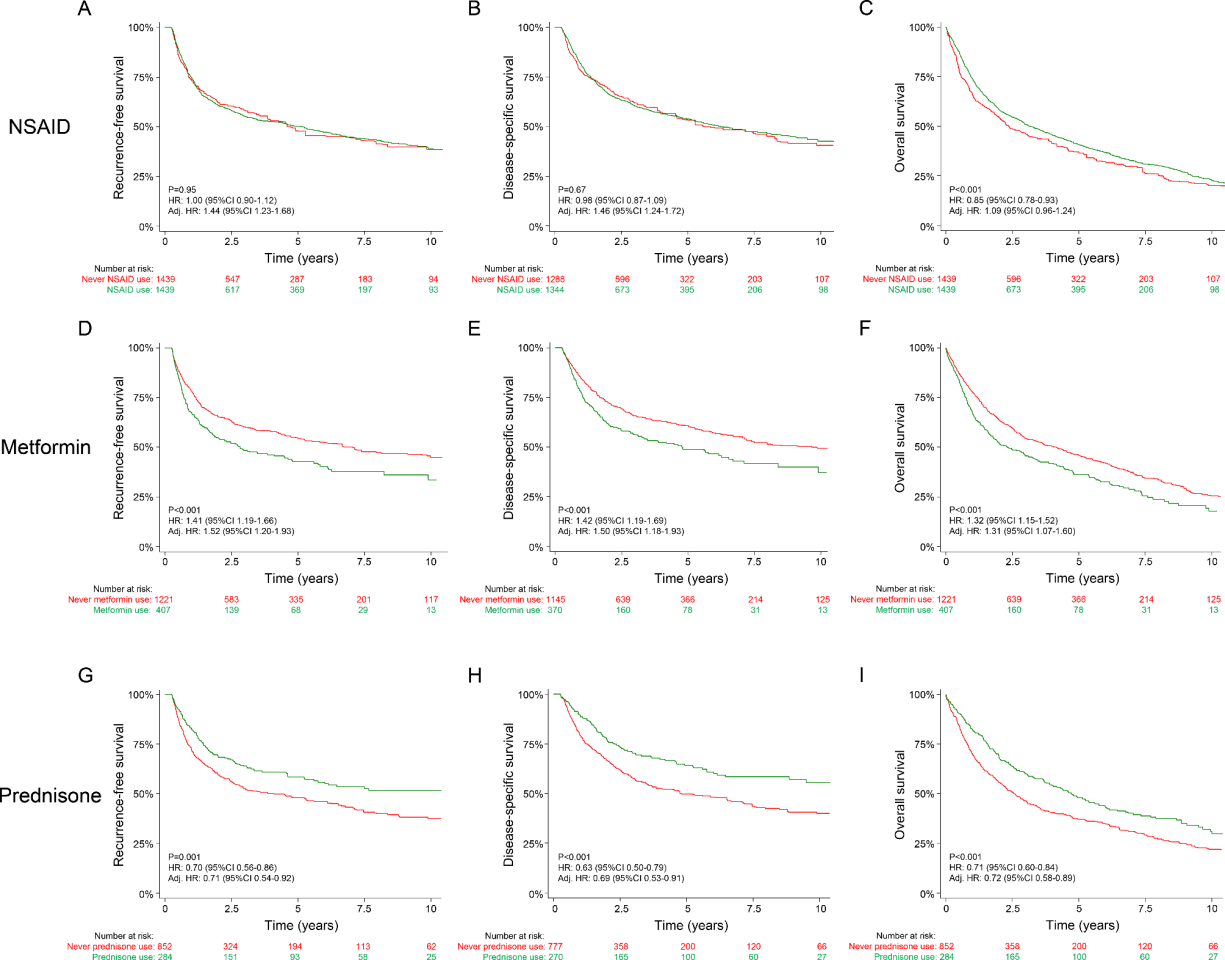
Clinical outcome of patients using NSAIDs, metformin, or prednisone before radical cystectomy Recurrence-free, disease-specific, and overall survival in patients who chronically used NSAIDs, metformin, or prednisone before radical cystectomy, as compared to a propensity-score matched cohort of never users. Hazard ratios (HR) and their respective 95% confidence intervals (95%CI) were calculated using univariable and multivariable Cox regression models. In multivariable analyses, hazard ratios were mutually adjusted for chronic preoperative statin, NSAID, metformin, and prednisone use NSAID, nonsteroidal anti-inflammatory drugs

Results were similar when analyzing our cohort using propensity-score matching (Fig. 1). Preoperative, chronic prednisone use remained significantly associated with improved recurrence-free, bladder cancer-specific, and overall survival, with adjusted HRs of 0.71 (95% CI 0.54–0.92), 0.69 (95% CI 0.53–0.91), and 0.72 (95% CI 0.58–0.89), respectively. These outcome parameters were significantly worse in metformin users, with adjusted HRs of 1.52 (95% CI 1.20–1.93), 1.50 (95% CI 1.18–1.93), and 1.31 (95% CI 1.07–1.60), respectively, and in NSAID users, with adjusted HRs of 1.44 (95% 1.23–1.68), 1.46 (95% CI 1.24–1.72), and 1.09 (95% CI 0.96–1.24). HRs were similar when separating NSAIDs by use of aspirin or other NSAIDs (supplementary Table 2).

Next, we evaluated whether medication use in the year following surgery was associated with clinical outcome. Baseline characteristics are depicted in supplementary Table 3 and were similar as in the preoperative chronic medication groups. In multivariable Cox proportional hazard models, directionality of adjusted HRs was the same in postoperative medication users as compared to preoperative, chronic medication users, but statistical power had decreased (Table 3). While postoperative statin use was still associated with clinical outcome, adjusted HRs for overall, bladder cancer-specific, and recurrence-free survival being 0.76 (95% CI 0.63–0.91), 0.65 (95% CI 0.53–0.81), and 0.69 (95% CI 0.56–0.84) respectively, postoperative prednisone use was not significantly associated with these outcome parameters, the adjusted HRs being 0.85 (95% CI 0.70–1.03), 0.87 (95% CI 0.69–1.09), and 0.87 (95% CI 0.71–1.08), respectively. Postoperative metformin use was only inversely associated with recurrence-free survival, with an adjusted HR of 1.31 (95% CI 1.01–1.70), while its association with overall survival (adjusted HR 1.18, 95% CI 0.93–1.50) and bladder cancer-specific survival (adjusted HR 1.32, 95% CI 0.99–1.75) was not significant. Postoperative NSAID use was still associated with a worse overall (adjusted HR 1.34, 95% CI 1.11–1.61), bladder cancer-specific (adjusted HR 1.46, 95% CI 1.18–1.82), and recurrence-free (adjusted HR 1.47, 95% CI 1.20–1.80) survival. This association was stronger in those using other NSAIDs postoperatively than in aspirin users.

**Table 3:** Characteristics associated with overall, bladder cancer-specific, and recurrence-free survival, by postoperative medication

	Overall survival		Bladder cancer-specific survival		Recurrence-free survival	
	Univariable	Multivariable	Univariable	Multivariable	Univariable	Multivariable
Postoperative medication use						
- Statins	0.90 (0.80–1.01)	**0.76** **(0.63–0.91)**	**0.79** **(0.69–0.91)**	**0.65** **(0.53–0.81)**	**0.82** **(0.72–0.93)**	**0.69** **(0.56–0.84)**
- NSAIDs	**1.14** **(1.00-1.30)**	**1.34** **(1.11–1.61)**	1.08 (0.92–1.27)	**1.46** **(1.18–1.82)**	1.12 (0.96–1.30)	**1.47** **(1.20–1.80)**
- Aspirin	**1.16** **(1.03–1.30)**	1.16 (0.96–1.41)	0.98 (0.85–1.14)	1.18 (0.94–1.48)	0.91 (0.80–1.04)	1.05 (0.85–1.30)
- Other NSAIDs	1.13 (0.99–1.29)	**1.25** **(1.05–1.49)**	**1.24** **(1.06–1.46)**	**1.49** **(1.22–1.82)**	**1.40** **(1.21–1.63)**	**1.55** **(1.29–1.87)**
- Metformin	1.16 (0.98–1.38)	1.18 (0.93–1.50)	1.19 (0.97–1.46)	1.32 (0.99–1.75)	**1.25** **(1.04–1.51)**	**1.31** **(1.01–1.70)**
- Prednisone	1.07 (0.92–1.23)	0.85 (0.70–1.03)	0.93 (0.77–1.11)	0.87 (0.69–1.09)	0.96 (0.81–1.13)	0.87 (0.71–1.08)

Uni- and multivariable Cox proportional-hazards regression models were used to calculate hazard ratios and their respective 95% confidence intervals. In the multivariable analyses, we adjusted for age, sex, region of residence, Charlson’s comorbidity index, year of surgery, distance to the hospital, hospital type, hospital and surgeon RC volume, type of bladder diversion, neoadjuvant chemotherapy use, and postoperative medication use (mutual adjustment for statin, NSAID, metformin, and prednisone use). Hazard ratios with a p-value of less than 0.050 are marked in bold

NSAID, nonsteroidal anti-inflammatory drugs; RC, radical cystectomy

## Discussion

In the past decades, literature on medication use and cancer outcome has been expanding. Many drugs that are used for other purposes than cancer may affect cancer growth. Due to the lack of randomized controlled trials, large cohort studies will provide the best level of evidence for determining associations between such medication use and cancer outcome. As such studies are subject to biases and confounding, it is important that findings are validated in independent studies. Therefore, we evaluated associations between chronic NSAID, metformin, and prednisone use, both prior to RC and in the year following RC, and bladder cancer outcome in a large cohort of bladder cancer patients undergoing RC in Québec province between 2000 and 2015.

This seems to be the first study reporting a positive association between prednisone use and clinical outcomes in bladder cancer patients. While the anti-tumor activity of glucocorticoids in other solid cancers, such as prostate, cervical, lung, and kidney cancers has been studied, literature on this medication in bladder cancer has been scarce and conflicting [18, 23]. A previous clinical study reported that reported that systemic glucocorticoid use increased (advanced) bladder cancer risk. A preclinical study conducted at Johns Hopkins University suggested that prednisone and other corticosteroids may suppress bladder tumor invasion without promoting cell proliferation [24]. The glucocorticoid receptor signaling pathway may directly be involved in bladder cancer progression, but it is also possible that prednisone affects bladder cancer outcome by interplay between the glucocorticoid receptor signaling pathway and sexual hormone receptor signaling such as the androgen and estrogen receptor pathways [18, 25]. Furthermore, inflammation plays a significant role in bladder cancer growth, which may be inhibited by prednisone [26]. Indeed, a recent study reported that the extent of inflammation in bladder cancer was a prognostic marker for overall survival in patients who underwent RC [27]. From this perspective, it would make sense that strong anti-inflammatory medication such as prednisone improves clinical outcome for bladder cancer patients. In our cohort, prednisone was prescribed to treat a variety of diseases, such as chronic obstructive pulmonary disease (COPD) and kidney diseases, and therefore, prednisone users were a heterogeneous cohort with potentially varying dosing levels and schedules. Urologists could have been more reluctant to conduct radical cystectomies on prednisone users who had advanced bladder cancer, although we adjusted for many covariates, including Charlson’s comorbidity index. Future clinical studies are needed to confirm our findings that pre- and/or postoperative prednisone use result in a survival benefit.

Metformin was not associated with improved bladder cancer outcome in our data; even more so, there seemed to be an inverse association. The worse outcome could indicate that metformin influences bladder cancer outcome. Alternatively, it may reflect previous findings that diabetes increases bladder cancer mortality [28]. When conducting a subgroup analysis with diabetic patients only, directionality remained the same, but metformin use was not significantly associated with a worse bladder cancer outcome anymore (data not shown), suggesting that at least part of the observed negative association between metformin and bladder cancer outcome was caused by the underlying disease and not the medication itself.

In preclinical studies, various anti-carcinogenic effects of metformin have been suggested, including its influence on insulin and glucose levels, immune-modulating effects of metformin, and inhibition of stem cell repopulation [31, 32]. A meta-analysis reported that metformin intake was associated with improved recurrence-free, progression-free, and cancer-specific survival, but not with overall survival [9]. However, these results were retrieved by combining data from much smaller clinical studies with very few cancer-related deaths as compared to our cohort. The inhibitory effect of metformin on bladder cancer should be regarded skeptically since no large cohort studies have confirmed such effect.

For NSAID use and bladder cancer outcome, the existing scientific literature is inconclusive. Daugherty et al. [29] concluded that nonaspirin NSAIDs, but not aspirin, may reduce bladder cancer risk. Similarly, a review in 2012 found no meaningful association between aspirin and bladder cancer [11]. Possibly, the anti-inflammatory effects of NSAIDs are smaller than those of prednisone, resulting in no significant effect on clinical outcome. However, other studies reported an association between aspirin use and improved bladder cancer outcome [7, 8, 30]. In our data, neither aspirin nor other NSAID users had an improved clinical outcome; outcomes were either similar or worse compared to never users. Intriguingly, outcomes were primarily worse after adjusting for other medication use. Hence, perhaps previously published studies that found an association between NSAID use and improved survival did not adjust well for comedication use. Alternatively, they could have been subject to publication bias or other biases/confounding. Either way, our data strengthen the suggestion that NSAIDs have no significant benefit for bladder cancer patients.

A major advantage of this study is that by collecting data from administrative databases in a publicly funded universal health care system, we did not restrict ourselves to certain hospitals or patient populations but included virtually all patients who underwent RC for bladder cancer in Québec province. However, selection bias may have occurred as we excluded patients who had private prescription drug insurance, and confounding by indication could obviously not be prevented. Nevertheless, biases were limited: e.g., to eliminate immortal time bias in our analyses of medication use post-surgery, we conducted a landmark analysis, limiting ourselves to patients who died ≥ 1 year after surgery while selecting patients based on medication use in the first year following surgery.

This study’s major limitation is that provincial registry data cannot be matched to clinical or pathological staging data; therefore, we were unable to evaluate whether cohorts differed in staging which would have affected the reported survival outcomes. RC is a complex surgery with significant postoperative morbidity and mortality [4]. Hence, it would be rational to be more reluctant conducting these surgeries in patients with severe comorbidity. This hypothesis is strengthened by the finding that a higher Charlson’s comorbidity index was associated with better bladder cancer-specific and recurrence-free survival. We also observed less neoadjuvant chemotherapy use in chronic NSAID or prednisone users, which may indicate that they less frequently had cT2-T4A tumors or received less aggressive therapy.

Other limitations have been described previously [4–6]. In short, missing variables may have resulted in residual confounding, such as smoking status. Misclassification bias may have occurred if patients used NSAIDs chronically but purchased the NSAID medication over-the-counter, which would not be registered in the provincial databases; such chronic NSAID users would have been misclassified as control patients. Additionally, restrictions in the dataset prevented us from calculating total years or total dose of medication use; we also do not know whether patients were therapy compliant. Finally, if patients moved out of the province, data collection stopped, including collection on death statistics. However, we did not expect patients with advanced bladder cancer to move frequently.

In conclusion, in our cohort of bladder cancer patients undergoing RC in Québec between 2000 and 2015, chronic prednisone use was associated with improved clinical outcomes, while chronic metformin and NSAID use were not. Future studies are needed to evaluate whether prednisone might be used to improve bladder cancer outcomes.

## Electronic supplementary material

Below is the link to the electronic supplementary material.

Additional File 1: Baseline characteristics, by preoperative chronic NSAID use

## Data Availability

The data that support the findings of this study are available from Québec provincial health authorities but restrictions apply to the availability of these data under Québec privacy laws, so are not publicly available. Data are however available from the authors upon reasonable request and with permission of CAI, RAMQ, MSSS, and ISQ.

## List of abbreviations

CAI: Commission d’accès à l’information du Québec; a committee enforcing protection of personal information including patient privacy in Québec province
CI: Confidence interval
COPD: Chronic obstructive pulmonary disease
HR: Hazard ratio
ISQ: Institut de la statistique du Québec; Québec province governmental organization for statistics, amongst others death statistics
MED-ÉCHO: A databank containing data on hospital stays in Quebec hospitals providing general and specialized care
MSSS: Ministère de la Santé et des Services sociaux (Ministry of Health and Social Services in Québec)
NSAID: Nonsteroidal anti-inflammatory drugs
RAMQ: Régie de l’assurance maladie du Québec; Québec province governmental organization which administers public health and drug insurance plans and pays health professionals
RC: Radical cystectomy
