# Supplementary material for: Chronic prednisone, metformin, and nonsteroidal anti-inflammatory drug use and clinical outcome in a cohort of bladder cancer patients undergoing radical cystectomy in Québec, Canada
Source: BMC Urol. 2023 Jul 14;23:119. doi: 10.1186/s12894-023-01287-6 (PMC10349444; doi:10.1186/s12894-023-01287-6)
Supplement: Supplementary file 1 — Additional File 1: Baseline characteristics, by preoperative chronic NSAID use [file 12894_2023_1287_MOESM1_ESM.docx]

**Supplementary table 1:** Baseline characteristics, by preoperative chronic NSAID use

|  | **Aspirin use** | | | **Use of other NSAIDs** | | |
| --- | --- | --- | --- | --- | --- | --- |
|  | **Preoperative chronic use** | **No** | **P-value** | **Preoperative chronic use** | **No** | **P-value** |
| Number of patients | 1144 | 1865 |  | 464 | 2036 |  |
| Age, median (IQR) | 73  (68-78) | 68  (61-74) | **<0.001** | 71  (64-75) | 71 (64-77) | **0.047** |
| Sex |  |  |  |  |  |  |
| - Male | 914  (79.9%) | 1336  (71.6%) | **<0.001** | 324  (69.8%) | 1568  (77.0%) | **0.001** |
| - Female | 230  (20.1%) | 529  (28.4%) |  | 140  (30.2%) | 468  (23.0%) |  |
| Region of residence |  |  |  |  |  |  |
| - Regions with cities with >400,000 inhabitants | 429  (37.5%) | 755  (40.5%) | 0.14 | 178  (38.4%) | 825  (40.5%) | 0.61 |
| - Regions with cities with 100,000-250,000 inhabitants | 629  (55.0%) | 982  (52.7%) |  | 256  (55.2%) | 1067  (52.4%) |  |
| - Rural regions (largest city in region <100,000 inhabitants) | 83  (7.3%) | 112  (6.0%) |  | 29  (6.3%) | 129  (6.3%) |  |
| - Unknown | 3  (0.3%) | 16  (0.9%) |  | 1  (0.2%) | 15  (0.7%) |  |
| Charlson’s comorbidity index, median (IQR) | 8  (6-9) | 6  (5-7) | **<0.001** | 7  (6-8) | 7  (5-8) | 0.23 |
| Year of surgery |  |  |  |  |  |  |
| - 2000-2009 | 606  (53.0%) | 1067  (57.2%) | **0.023** | 293  (63.1%) | 1128  (55.4%) | **0.002** |
| - 2010-2014 | 538  (47.0%) | 798  (42.8%) |  | 171  (36.9%) | 908  (44.6%) |  |
| Hospital characteristics |  |  |  |  |  |  |
| Distance to hospital in km, median (IQR) | 20  (7-88) | 20  (7-65) | 0.43 | 20  (7-77) | 18  (7-70) | 0.54 |
| Hospital type, academic | 554  (48.4%) | 970  (52.0%) | 0.06 | 235  (50.6%) | 1021  (50.1%) | 0.85 |
| Hospital RC volume/year, median (IQR) | 13  (8-32) | 13  (8-32) | 0.43 | 13  (6-32) | 13  (8-32) | 0.17 |
| Surgeon RC volume/year, median (IQR) | 7  (5-13) | 7  (5-13) | 0.93 | 7  (5-13) | 7  (5-13) | 0.45 |
| Type of bladder diversion |  |  |  |  |  |  |
| - Ileal conduit | 986  (86.2%) | 1477  (79.2%) | **<0.001** | 379  (81.7%) | 1668  (81.9%) | 0.97 |
| - Continent diversion | 115  (10.1%) | 312  (16.7%) |  | 66  (14.2%) | 289  (14.2%) |  |
| - Unknown | 43  (3.8%) | 76  (4.1%) |  | 19  (4.1%) | 79  (3.9%) |  |
| Neoadjuvant chemotherapy | 51  (4.5%) | 136  (7.3%) | **0.002** | 25  (5.4%) | 121  (5.9%) | 0.65 |
| Preoperative chronic medication use |  |  |  |  |  |  |
| - Statins | 836  (73.1%) | 377  (20.2%) | **<0.001** | 197  (42.5%) | 771  (37.9%) | 0.17 |
| - NSAIDs | 1144  (100%) | 213  (11.4%) | **<0.001** | 464  (100%) | 624  (30.6%) | **<0.001** |
| - Aspirin | - | - | - | 166  (35.8%) | 624  (30.6%) | 0.09 |
| - Other NSAIDs | 166  (14.5%) | 213  (11.4%) | **0.031** | - | - | - |
| - Metformin | 257  (22.5%) | 101  (5.4%) | **<0.001** | 56  (12.1%) | 243  (11.9%) | 0.46 |
| - Prednisone | 116  (10.1%) | 115  (6.2%) | **<0.001** | 60  (12.9%) | 131  (6.4%) | **<0.001** |
| Follow-up time (in years), median (95% CI) | 6.8  (6.3-7.4) | 7.1  (6.6-7.6) |  | 8.7  (7.6-10.0) | 7.3  (6.6-7.7) |  |

CI, confidence interval; IQR, interquartile range; NSAID, Nonsteroidal anti-inflammatory drugs; RC, radical cystectomy.

Significant p-values (P<0.050) are in bold.

**Supplementary table 2:** Chronic preoperative aspirin or other nonsteroidal anti-inflammatory drugs (NSAID) use and overall, bladder cancer-specific and recurrence-free survival.

|  | **Overall survival** | | **Bladder cancer-specific survival** | | **Recurrence-free survival** | |
| --- | --- | --- | --- | --- | --- | --- |
|  | **Univariable** | **Multivariable** | **Univariable** | **Multivariable** | **Univariable** | **Multivariable** |
| **Full cohort** | | | | | | |
| Aspirin | 1.04  (0.95-1.14) | 1.08  (0.93-1.26) | 0.95  (0.84-1.06) | 1.09  (0.90-1.31) | 0.88  (0.79-0.98) | 1.01  (0.84-1.21) |
| Other NSAIDs | **0.86**  **(0.76-0.97)** | 0.92  (0.78-1.09) | 0.93  (0.80-1.09) | 1.11  (0.91-1.35) | 1.02  (0.88-1.18) | 1.16  (0.96-1.40) |
| **Propensity-score matched cohort** | | | | | | |
| Aspirin | **0.90**  **(0.81-0.99)** | 1.14  (0.98-1.32) | 0.90  (0.79-1.02) | 1.15  (0.95-1.38) | **0.88**  **(0.78-1.00)** | 1.10  (0.91-1.31) |
| Other NSAIDs | 0.96  (0.82-1.14) | 0.88  (0.71-1.10) | 1.04  (0.85-1.28) | 1.06  (0.82-1.37) | 1.20  (0.99-1.46) | 1.20  (0.93-1.54) |

Uni- and multivariable Cox proportional-hazards regression models were used to calculate hazard ratios and their respective 95% confidence intervals. Hazard ratios with a p-value of less than 0.050 are marked in bold.

In the multivariable analyses of the full cohort, aspirin/other NSAID use was adjusted for preoperative chronic statin, metformin and prednisone use, age, sex, region of residence, Charlson’s comorbidity index, year of surgery, distance between a patient’s residence and the hospital of radical cystectomy, hospital type (academic/non-academic), hospital and surgeon radical cystectomy volume, type of bladder diversion, and neoadjuvant chemotherapy use.

In the propensity-score matching, never-aspirin users were matched 1:1 by age, sex, region of residence, Charlson’s comorbidity index, year of surgery, distance between a patient’s residence and the hospital of radical cystectomy, hospital type (academic/non-academic), hospital and surgeon radical cystectomy volume, type of bladder diversion, and neoadjuvant chemotherapy use, using a caliper width of 0.2. In its multivariable analyses, we adjusted for statin, metformin and prednisone use.

**Supplementary table 3:** Baseline characteristics, by postoperative medication use.

|  | **All patients** | **NSAID use** | | | **Metformin use** | | | **Prednisone use** | | |
| --- | --- | --- | --- | --- | --- | --- | --- | --- | --- | --- |
|  |  | **Postoperative use** | **No** | **P-value** | **Postoperative use** | **No** | **P-value** | **Postoperative use** | **No** | **P-value** |
| Number of patients | 2676 | 1266 | 667 |  | 291 | 2173 |  | 416 | 1559 |  |
| Age, median (IQR) | 69  (63-75) | 71  (66-76) | 67  (60-74) | **<0.001** | 71  (66-75) | 69  (62-75) | **0.010** | 71  (66-76) | 68  (61-74) | **<0.001** |
| Sex |  |  |  |  |  |  |  |  |  |  |
| - Male | 2025 (75.7%) | 963  (76.1%) | 493  (73.9%) | 0.30 | 237  (81.4%) | 1619  (74.5%) | **0.010** | 295  (70.9%) | 1224  (78.5%) | **0.001** |
| - Female | 651  (24.3%) | 303  (23.9%) | 174  (26.1%) |  | 54  (18.6%) | 554  (25.5%) |  | 121  (29.1%) | 335  (21.5%) |  |
| Region of residence |  |  |  |  |  |  |  |  |  |  |
| - Regions with cities with >400,000 inhabitants | 1049  (39.2%) | 489  (38.6%) | 286  (42.9%) | 0.06 | 123  (43.3%) | 846  (38.9%) | 0.53 | 151  (36.3%) | 625  (40.1%) | 0.16 |
| - Regions with cities with 100,000-250,000 inhabitants | 1439  (53.8%) | 690  (54.5%) | 345  (51.7%) |  | 148  (50.9%) | 1178  (54.2%) |  | 231  (55.5%) | 831  (53.3%) |  |
| - Rural regions (largest city in region <100,000 inhabitants) | 172  (6.4%) | 82  (6.5%) | 30  (4.5%) |  | 18  (6.2%) | 135  (6.2%) |  | 33  (7.9%) | 92  (5.9%) |  |
| - Unknown | 16  (0.6%) | 5  (0.4%) | 6  (0.9%) |  | 2  (0.7%) | 14  (0.6%) |  | 1  (0.2%) | 11  (0.7%) |  |
| Charlson’s comorbidity index, median (IQR) | 7  (5-8) | 7  (6-9) | 6  (5-7) | **<0.001** | 8  (7-10) | 7  (5-8) | **<0.001** | 7  (6-9) | 6  (5-8) | **<0.001** |
| Year of surgery |  |  |  |  |  |  |  |  |  |  |
| - 2000-2009 | 1602  (59.9%) | 742  (58.6%) | 356  (53.4%) | **0.027** | 129  (44.3%) | 1329  (61.2%) | **<0.001** | 243  (58.4% | 851  (54.6%) | 0.16 |
| - 2010-2015 | 1074  (40.1%) | 524  (41.4%) | 311  (46.6%) |  | 162  (55.7%) | 844  (38.8%) |  | 173  (41.6%) | 708  (45.4%) |  |
| Hospital characteristics |  |  |  |  |  |  |  |  |  |  |
| Distance to hospital in km, median (IQR) | 20  (7-71) | 20  (7-80) | 16  (7-50) | **0.024** | 21  (8-78) | 19  (7-66) | 0.33 | 23  (9-80) | 19  (7-70) | 0.07 |
| Hospital type, academic | 1351  (50.5%) | 623 (49.2%) | 353 (52.9%) | 0.12 | 143  (49.1%) | 1103  (50.8%) | 0.60 | 231  (55.5%) | 794  (50.9%) | 0.10 |
| Hospital RC volume/year, median (IQR) | 13  (8-32) | 13  (8-32) | 13  (8-32) | 0.51 | 13  (7-32) | 13  (8-32) | 0.69 | 14  (7-32) | 13  (9-32) | 0.65 |
| Surgeon RC volume/year, median (IQR) | 7  (5-13) | 7  (5-13) | 8  (5-13) | 0.36 | 7  (5-10) | 7  (5-13) | 0.85 | 9  (5-13) | 8  (5-13) | 0.15 |
| Type of bladder diversion |  |  |  |  |  |  |  |  |  |  |
| - Ileal conduit | 2109  (78.8%) | 1031 (81.4%) | 522  (78.3%) | **0.025** | 243  (83.5%) | 1699  (78.2%) | 0.09 | 340  (81.7%) | 1197  (76.8%) | **0.013** |
| - Continent diversion | 466  (17.4%) | 184  (14.5%) | 124  (18.6%) |  | 41  (14.1%) | 386  (17.8%) |  | 61  (14.7%) | 314  (20.1%) |  |
| - Unknown | 101  (3.8%) | 51  (4.0%) | 21  (3.1%) |  | 7  (2.4%) | 88  (4.0%) |  | 15  (3.6%) | 48  (3.1%) |  |
| Neoadjuvant chemotherapy | 146  (5.5%) | 51 (4.0%) | 54 (8.1%) | **<0.001** | 15  (5.2%) | 118  (5.4%) | 0.85 | 15  (3.6%) | 106  (6.8%) | **0.016** |
| Postoperative medication use |  |  |  |  |  |  |  |  |  |  |
| - Statins | 1064  (39.8%) | 751  (59.3%) | 146 (21.9%) | **<0.001** | 218  (74.9%) | 761  (35.0%) | **<0.001** | 188  (45.2%) | 593  (38.0%) | **0.022** |
| - NSAIDs | 1266  (47.3%) | - | - | - | 197  (67.7%) | 973  (44.8%) | **<0.001** | 250  (60.1%) | 660  (42.3%) | **<0.001** |
| - Aspirin | 884  (33.0%) | 884 (69.8%) | - | - | 172  (59.1%) | 638  (29.4%) | **<0.001** | 172  (41.3%) | 457  (29.3%) | **<0.001** |
| - Other NSAIDs | 537  (20.1%) | 537 (42.4%) | - | **-** | 66  (22.7%) | 436  (20.1%) | 0.30 | 120  (28.8%) | 273  (17.5%) | **<0.001** |
| - Metformin | 291  (10.9%) | 197  (15.6%) | 45  (6.7%) | **<0.001** | - | - | - | 50  (12.0%) | 166  (10.6%) | 0.33 |
| - Prednisone | 416  (15.5%) | 250  (19.7%) | 68  (10.2%) | **<0.001** | 50  (17.2%) | 341  (15.7%) | 0.80 | - | - | - |

IQR, interquartile range; NSAID, nonsteroidal anti-inflammatory drugs; RC, radical cystectomy.

Patients with a survival less than 1 year were excluded, to remove immortal time bias.

Significant p-values (P<0.050) are in bold.
